# Evaluating *apolipoprotein E* genotype status and neuroprotective effects against white matter hyperintensity development in high-altitude careers

**DOI:** 10.1186/s13104-018-3867-7

**Published:** 2018-10-25

**Authors:** Richard R. Chapleau, CharLee A. Martin, Summer R. Hughes, James C. Baldwin, John Sladky, Paul M. Sherman, Michael Grinkemeyer

**Affiliations:** 1 US Air Force School of Aerospace Medicine, Aeromedical Research Department, Applied Technology and Genomics Division, Wright-Patterson AFB, Dayton, OH US; 2 US Air Force School of Aerospace Medicine, Aeromedical Research Department, Operational Health and Performance Research Division, Wright-Patterson AFB, Dayton, OH US; 3 59th Medical Wing, Department of Neurology, Joint Base San Antonio-Lackland, Houston, TX US; 4 59th Medical Wing, Department of Neuroradiology, Joint Base San Antonio-Lackland, Houston, TX US

**Keywords:** Apolipoprotein E, High altitude acclimatization, Neuroprotection, Genotype screening

## Abstract

**Objective:**

This study considers the use of a rapid molecular assay to evaluate *apolipoprotein E* (*ApoE*) status in military subjects who have been exposed to high altitude. We hypothesize that *ApoE* status may be protective against developing brain white matter hyperintensities (WMHs) after high altitude exposure.

**Results:**

We tested 92 subjects who had been exposed to altitudes above 25,000 ft mean sea level, either as pilots or as altitude chamber technicians. We determined subject genetic status using rapid Taqman-style polymerase chain reaction genotyping and evaluated the association of *ApoE* subtype versus brain lesions using t-tests and two-way analyses of variance. Our results indicate that there is no significant association between *ApoE* genotype status and the presence of WMHs after high altitude exposure. We did observe a significantly higher number of hours spent at altitude for subjects with the *ApoE E2* allele; however, the mechanism by which this may occur is not determined in this study. To more fully elucidate this effect, larger populations would be required to observe greater numbers of subjects with the *E2* and *E4* alleles.

## Introduction

Genetic variants of the a*polipoprotein E* (*ApoE*) gene have been associated with several neurodegenerative disorders including Alzheimer’s disease, cognitive impairment, and multiple sclerosis, as well as with varying rates of recovery from traumatic brain injury [1–3]. In the brain, non-neuronal cell types, most notably astroglia and microglia, are the primary producers of ApoE proteins, while neurons preferentially express the receptors for ApoE.

The *ApoE4* genotype, specifically, is associated with an increased risk of developing Alzheimer’s disease (20% in controls vs. 90% in *ApoE4* patients) [4, 5] and a decreased average age of onset from 84 to 68 years, respectively [4]. The mechanism by which *ApoE4* participates in disease pathogenesis is not known. Paradoxically, the same genotype is associated with increased neuroprotection against Alzheimer’s disease provided by consumption of fish [6].

In a meta-analysis of 42 published studies conducted by Schilling et al. the *ApoE4* and *ApoE2* genes were associated with increasing burden in magnetic resonance imaging (MRI) markers for both hemorrhagic and ischemic cerebrovascular disease, while *ApoE2* correlated with increasing brain white matter hyperintensities (WMHs) [7]. In previous studies from our group [8, 9], increased WMH burden was associated with repetitive non-hypoxic hypobaric exposure, neurologic decompression sickness, and lower neurocognitive test performance as measured on computer-based neurocognitive tests. High altitude aviators are at risk for decompression sickness, with an anonymous survey noting a self-reported prevalence of 75.5% [10].

Because these lesions are destructive to the normal brain architecture, WMHs may be considered a form of traumatic brain injury. In high altitude exposed individuals, these lesions are presumed to be secondary to repetitive hypobaric exposure exacerbated by an increased operation tempo and increased workload during exposure. Determining the etiology of WMHs may lead to important mitigation strategies for high altitude operators. We hypothesized that *ApoE* status may be a contributing risk factor in developing WMHs after high altitude exposures.

## Main text

### Methods

#### Study design and MRI data collection

We recruited high altitude aviators from both pilot (n = 44) and altitude chamber operator career fields (n = 48). All subjects were male and had brain MRI results obtained from a previous study. All participants were healthy and were selected for the MRI as a consequence of career selection, not as a result of clinical complaint. This study was approved by the Air Force Research Laboratory’s Institutional Review Board (FWR20160040H).

#### Sample collection and laboratory analysis

We provided consenting subjects with blood tubes and directed them to report to their local medical treatment facility for phlebotomy. Blood samples were sent from the treatment facility to our research laboratory for processing. Nucleic acids were extracted using the Promega Maxwell 16 Blood DNA Purification kit and we obtained *ApoE* genotype status by performing two polymerase chain reaction genotyping assays (rs7412 and rs429358). Commercially produced Taqman-style assays were purchased directly from Thermo Fisher and the assays were performed on an Applied Biosystems 7500 FAST real-time thermocycler. The two genotyping assays were verified using 10 control samples obtained and pretested at a reference lab.

#### Statistical analysis

We evaluated the association between *ApoE* genotype status and each of the phenotype variables using GraphPad Prism 7.0c. Two-way analysis of variance was performed with genotype as a primary factor and the phenotypes as the other contributing factors. Parameters for the multiple t-tests performed included the assumption of similar scatter and a 10% false discovery rate using the two-stage step-up method of Benjamini et al. [11]. Descriptive statistics were also obtained in Prism 7, including the mean, median, deviations, and ranges.

### Results

Overall *ApoE* allele frequencies were consistent with the global allele distribution [12]: *ApoE2 *= 7.6%, *ApoE3 *= 78.8%, and *ApoE4 *= 13.5%. These alleles were spread across four genotypes: *ApoE2/ApoE3, ApoE3/ApoE3, ApoE3/ApoE4,* and *ApoE4/ApoE4*. Performing a two-way analysis of variance revealed that the *ApoE* genotype status accounted for 0.21% of the total variance in the population, the interaction between genotype and collected phenotypes accounted for 1.99%, and the differences in the phenotypes themselves accounted for 16.92% of the variance. The effects of both the interaction and the genotype are not considered significant. Additionally, performing multiple t-tests to evaluate possible associations between genotype and WHM lesion count or volume revealed no significant associations (Table 1).

**Table 1:** Statistical analysis of multiple t-tests comparing genotype versus WMH lesions

Genotype comparison	WMH count p-value	WMH volume p-value	Genotype comparison	WMH count p-value	WMH volume p-value
*ApoE2/ApoE3* vs. *ApoE3/ApoE3*	0.92	> 0.99	*ApoE3/ApoE3* vs. *ApoE3/ApoE4*	0.95	> 0.99
*ApoE2/ApoE3* vs. *ApoE3/ApoE4*	0.97	> 0.99	*ApoE3/ApoE3* vs. *ApoE4/ApoE4*	0.93	> 0.99
*ApoE2/ApoE3* vs. *ApoE4/ApoE4*	0.97	> 0.99	*ApoE3/ApoE4* vs. *ApoE4/ApoE4*	0.95	> 0.99

One interesting result discovered was a positive association between genotype and hours flown above 25,000 ft mean sea level (Table 2). The subjects with the *ApoE2/ApoE3* genotype had significantly more hours above this altitude than those with the *ApoE*3/*ApoE3* or *ApoE3*/*ApoE4* genotypes (p-values of 1.1e−9 and 6.2e−6, respectively). There was no significant association discovered for *ApoE2/ApoE3* or *ApoE4/ApoE4*. However, since there were only two subjects with the latter genotype, no statistical inferences can be made. Looking further into the flight hour details uncovered a wide range of values for the test population. The range of times for the entire population was from 9 to 2000 h.

**Table 2:** Range of accumulated exposures above 25,000 ft mean sea level by genotype

Genotype	Median	Upper	Lower	Mean	Deviation (%)
*ApoE2/ApoE3*	490.5	1630	21	631.4	527.5
*ApoE3/ApoE3*	139	2000	9	387.2	461.0
*ApoE3/ApoE4*	202	1700	12	405.7	455.2
*ApoE4/ApoE4*	665	1157	173	665	695.8

### Discussion

As more high performance aircraft are being developed with service ceilings greater than 50,000 ft, it is becoming increasingly important to understand the impact of altitude on human performance. In addition to high altitude aviators and operators (including military freefall parachutists and aerospace physiologists), the identification of risk factors for altitude-induced illness could influence decisions of adventurers, travelers, and even civilians moving to or living in mountainous regions. Additionally, members of the commercial airline industry may benefit from a greater understanding of the relationship between altitude, disease, and genetics.

Although our hypothesis was disproven by our results, we feel that this study provides critical information in that at least one of the genetic markers related to neurodegeneration is not an apparent risk factor for altitude-induced brain injury. There may be a protective effect of *ApoE* status that contributes to a significantly higher number of hours spent at altitude for subjects with the *ApoE2* allele; however, the mechanism by which this may occur was not determined in this study. To more fully elucidate this effect, larger populations would be required to observe greater numbers of subjects with the *ApoE2* and *ApoE4* alleles. Our subject population includes a representative sampling of between 50% and 90% of currently qualified high altitude pilots [13], and approximately 1% of the total population of high altitude pilots ever to have flown. Therefore, our results are expected to highly reflect the true population.

Another alternative to identify if *ApoE* is implicated in WMH development would be to assess polymorphisms in the gene’s promoter region. To that end, we are currently exploring the hypothesis that there is an association between either variant rs405509 (-219G/T) or rs769446 (-427T/C) and WMH development. These variants have been associated in the past with brain functional decline and may be a confounding factor in altitude-induced injury [14, 15]. We anticipate developing a better understanding of *ApoE*’s role in altitude-related negative effects through the currently reported results regarding *ApoE* status and the up-coming work on promoter genotypes.

## Limitations

This study focused on a single gene and had a small sample size with widely varying phenotype data. Additional studies of larger numbers of subjects with high altitude exposure may provide further insight into possible genetic risk factors for altitude-induced neurodegenerative outcomes. Alternative hypothesis testing of additional genes, and/or larger studies with genome-wide technologies, may identify relevant markers.

## Abbreviations

*ApoE*: *apolipoprotein E*
MRI: magnetic resonance imaging
WMH: white matter hyperintensity
